# What does soil-transmitted helminth elimination look like? Results from a targeted molecular detection survey in Japan

**DOI:** 10.1186/s13071-019-3875-z

**Published:** 2020-01-08

**Authors:** Mitsuko Hasegawa, Nils Pilotte, Mihoko Kikuchi, Arianna R. Means, Marina Papaiakovou, Andrew M. Gonzalez, Jacqueline R. M. A. Maasch, Hiroshi Ikuno, Toshihiko Sunahara, Kristjana H. Ásbjörnsdóttir, Judd L. Walson, Steven A. Williams, Shinjiro Hamano, the DeWorm3 Project Team

**Affiliations:** 1 Department of Parasitology, Institute of Tropical Medicine (NEKKEN), Nagasaki University, Nagasaki, Japan; 2 Doctoral Leadership Program, Graduate School of Biomedical Sciences, Nagasaki University, Nagasaki, Japan; 3 The Joint Usage/Research Center on Tropical Disease, Institute of Tropical Medicine (NEKKEN), Nagasaki University, Nagasaki, Japan; 4 Department of Biological Sciences, Smith College, Northampton, Massachusetts USA; 5 Molecular and Cellular Biology Program, University of Massachusetts, Amherst, Massachusetts USA; 6 Department of Immunogenetics, Institute of Tropical Medicine (NEKKEN), Nagasaki University, Nagasaki, Japan; 7 Department of Global Health, University of Washington, Seattle, Washington USA; 8 Department of Life Sciences, Natural History Museum, London, UK; 9 Department of Bacteriology, BML, Inc, Saitama, Japan; 10 Department of Vector Ecology and Environment, Institute of Tropical Medicine (NEKKEN), Nagasaki University, Nagasaki, Japan; 11 Department of Epidemiology, University of Washington School of Public Health, Seattle, Washington USA; 12 Departments of Medicine (Infectious Disease), Pediatrics and Epidemiology, University of Washington, Seattle, Washington USA

**Keywords:** Soil-transmitted helminth, STH, *Ascaris lumbricoides*, *Ancylostoma duodenale*, *Necator americanus*, *Trichuris trichiura*, Targeted prevalence survey, Multi-parallel real-time PCR, WASH

## Abstract

**Background:**

Japan is one of the few countries believed to have eliminated soil-transmitted helminths (STHs). In 1949, the national prevalence of *Ascaris lumbricoides* was 62.9%, which decreased to 0.6% in 1973 due to improvements in infrastructure, socioeconomic status, and the implementation of national STH control measures. The Parasitosis Prevention Law ended in 1994 and population-level screening ceased in Japan; therefore, current transmission status of STH in Japan is not well characterized. Sporadic cases of STH infections continue to be reported, raising the possibility of a larger-scale recrudescence of STH infections. Given that traditional microscopic detection methods are not sensitive to low-intensity STH infections, we conducted targeted prevalence surveys using sensitive PCR-based assays to evaluate the current STH-transmission status and to describe epidemiological characteristics of areas of Japan believed to have achieved historical elimination of STHs.

**Methods:**

Stool samples were collected from 682 preschool- and school-aged children from six localities of Japan with previously high prevalence of STH. Caregivers of participants completed a questionnaire to ascertain access to water, sanitation and hygiene (WASH), and potential exposures to environmental contamination. For fecal testing, multi-parallel real-time PCR assays were used to detect infections of *Ascaris lumbricoides*, *Necator americanus*, *Ancylostoma duodenale* and *Trichuris trichiura*.

**Results:**

Among the 682 children, no positive samples were identified, and participants reported high standards of WASH.

**Conclusions:**

To our knowledge, this is the first STH-surveillance study in Japan to use sensitive molecular techniques for STH detection. The results suggest that recrudescence of STH infections has not occurred, and that declines in prevalence have been sustained in the sampled areas. These findings suggest that reductions in prevalence below the elimination thresholds, suggestive of transmission interruption, are possible. Additionally, this study provides circumstantial evidence that multi-parallel real-time PCR methods are applicable for evaluating elimination status in areas where STH prevalence is extremely low.
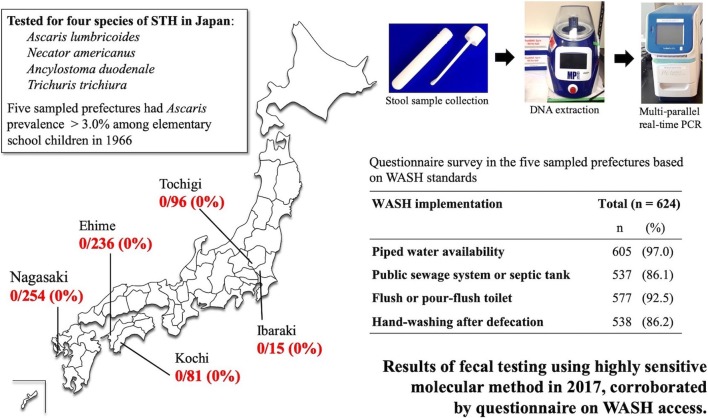

## Background

Almost two billion people, or nearly one quarter of the total global population, are infected with one or more soil-transmitted helminth (STH) species, including *Ascaris lumbricoides*, hookworm (*Ancylostoma duodenale* and *Necator americanus*) and *Trichuris trichiura* [1, 2]. Although individuals with low-intensity STH infections may be asymptomatic, heavy infections are associated with considerable morbidity, including abdominal pain, diarrhea and anemia, as well as impaired cognitive and physical development in children [3]. Additionally, STH infections lead to considerable loss of healthy life-years lived, as measured by the disability-adjusted life-years (DALYs) metric [4]. STH-related global DALYs were estimated at 1.92 million in 2017 [5].

Current World Health Organization (WHO) guidelines for the management of STH infections focus on morbidity control through the use of mass drug administration (MDA) of anthelmintic drugs targeting preschool- and school-aged children as well as other at-risk groups [1]. Although this strategy can be successful in reducing the burden of high-intensity STH infections (those that result in morbidity) within a population, targeted MDA alone likely does not eliminate these infections, as reinfection remains common in treated communities. One reason for high reinfection rates is that a large segment of the population, including most adults, remains untreated and serves as reservoirs for continued transmission. As a result, morbidity control programmes may need to be continued indefinitely in some settings or at least until economic development and improved sanitation practices can prevent ongoing transmission [6, 7]. Mathematical models suggest that MDA programmes targeting community members of all ages may interrupt STH transmission [8]. As a result, there is interest in determining whether STH infections can be eliminated in focal geographical settings, with a number of ongoing studies attempting to address this question [9–11].

**Fig. 1:**
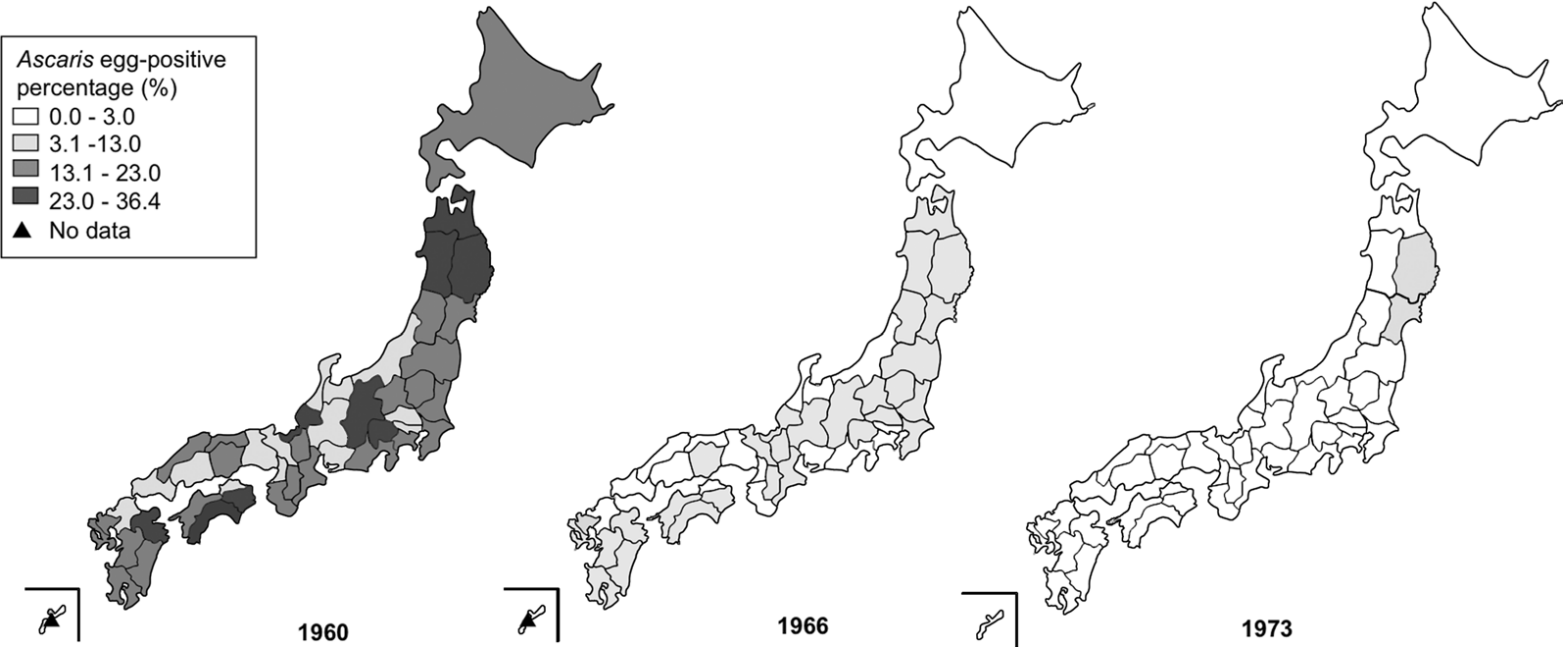
Historical trend map of *Ascaris* egg-positive percentage among elementary school children. *Ascaris* egg-positive percentages in 1960, 1966 and 1973 are represented in gray scale. These maps were created using statistical data from the Ministry of Education, Culture, Sports, Science and Technology, Japan [18–20] and the website Hakuchizu nurinuri (https://n.freemap.jp), which provides copyright-free maps. The maps were then edited with Adobe Photoshop version CS 5.1

It is important to determine whether elimination of STH transmission has truly been achieved in areas considered to have successfully eliminated human STH infections, such as in Japan [12, 13]. In 1949 (after the World War II), the national prevalence of *Ascaris* was estimated at 62.9% among people of all ages in Japan [14]. Therefore, based on the Parasitosis Prevention Law enacted in 1931 for prevention of ascariasis, ancylostomiasis, schistosomiasis and clonorchiasis, routine mass screening, selective drug administration, night-soil treatment, and health education were implemented nationwide to treat and prevent STH infections [15–17]. As a result, and along with improvements in national socioeconomic standards and improved water and sewage infrastructure, national *Ascaris* prevalence plummeted over the following two decades, reaching 0.6% in 1973 [15]. In the same year, the School Health and Safety Act (named the School Health Act at the time) was amended to exclude children aged ≥ 12 years from mandatory stool tests for STH eggs. The act was further amended to not include children aged ≥ 9 years in 1994 and eventually not require stool tests for STH for any child in 2002. National-level and sub-national-level statistics regarding STH prevalence have not been available since 1973 (Fig. 1) [18–20]. The Japan Association of Parasite Control (JAPC) continued fecal testing services in Japan for the detection of parasite eggs, even after 1973; however, since repeal of the Parasitosis Prevention Law in 1994, screening activities conducted by JAPC have decreased dramatically [21].

Although it is generally accepted that STH transmission has been interrupted in Japan, the actual status of STH transmission has not been systematically evaluated, and sporadic cases of STH continue to be reported in Japan by JAPC and clinical institutions [22–26] (Table 1). Although many of these infections are potentially acquired overseas [27–29] or *via* consumption of contaminated imported food [30–32], these data suggest that limited transmission of STH infection could still be occurring in Japan. In the USA, also thought to have interrupted STH transmission [33], a recent survey conducted in rural Alabama using real-time PCR demonstrated that 19 of 55 individuals tested were positive for *N. americanus*, indicating continued focal transmission [34]. Limited numbers of STH cases could potentially lead to a larger-scale recrudescence of STH transmission in the future; therefore, prevalence surveys to evaluate the current transmission status of STH in Japan are necessary and can inform the need for continued STH surveillance following the success of control programmes.

**Table 1:** Number of detected STH cases in Japanese nationals according to data from BML, Inc

Year	STH total	*Trichuris*	Hookworm	*Ascaris*
2000	62 (2)^ab^	23 (1)^a^	20 (1)^b^	19 (2)^ab^
2001	76	24	25	27
2002	93	29	33	31
2003	48	17	9	22
2004	41	13	9	19
2005	37	9	9	19
2006	21	4	0	17
2007	19 (1)^a^	7 (1)^a^	0	12 (1)^a^
2008	26	10	5	11
2009	13	8	2	3
2010	16	7	2	7
2011	9	7	1	1
2012	22	13	2	7
2013	5	3	0	2
2014	9	6	0	3
2015	5	3	1	1
2016	4	2	1	1
2017	6	6	0	0
Total	512 (3)^c^	191 (2)^c^	119 (1)^c^	202 (3)^c^

*Notes*: The numbers in parentheses denote individuals with dual infection. Tween 80 citric acid ether sedimentation was used to detect STH eggs [26]. BML, Inc. is a privately-owned clinical laboratory company based in Tokyo, Japan. The data are courtesy of BML

^a^*Trichuris* + *Ascaris* co-infections

^b^Hookworm + *Ascaris* co-infections

^c^Total number of individuals with co-infections

In this study, we conducted targeted prevalence surveys in areas previously reporting high STH prevalence in Japan. Fecal samples were tested with multi-parallel real-time PCR assays, a highly sensitive and specific molecular method [35], to evaluate the current state of STH transmission in preschool- and school-aged children in Japan. Additionally, we retrospectively evaluated the cumulative effects of decades of Japanese STH-control measures, including large-scale screenings with selective drug administration, as well as improved access to water, sanitation and hygiene (WASH) [36]. Questionnaire surveys were conducted to determine risk factors of STH infection and ascertain WASH implementation. Fecal samples and filled questionnaires were collected from elementary schools, kindergartens and a nursery, within five participating prefectures (Nagasaki, Ehime, Kochi, Tochigi and Ibaraki) in Japan. The results of these targeted prevalence surveys were used to describe the epidemiological characteristics of areas believed to have achieved historical elimination of STHs.

## Methods

### Site selection

For fecal sampling in this study, we selected prefectures that met the following two conditions: (i) those with *Ascaris* prevalence > 3.0% among elementary school children in 1966 (Fig. 1) [19]; and (ii) those with at least one confirmed STH case within a 15-year time-frame from 2002 to 2016. We used the *Ascaris* prevalence in elementary school boys or girls (whichever was higher) as a representative indicator in each prefecture, because government data provided only the average *Ascaris* percentages for boys and girls separately by prefecture. STH case-number data from BML, Inc., a privately owned clinical laboratory company based in Tokyo, Japan, and from JAPC were reviewed to identify prefectures for inclusion in this study (Additional file 1: Tables S1, S2) [37–45]. The sampling frame for participating schools was limited to 23 of 47 prefectures based on the inclusion criteria (Additional file 2: Figure S1). The authors contacted the municipal education boards and relevant school administrators to seek participation if a prefecture met the inclusion criteria. Schools were then selected from these prefectures based on willingness to participate in the stool surveys.

### Target population

All preschool- and school-aged children between 2–14 years of age [1] and attending the selected schools were invited to participate in this study. Potential participants were excluded if they did not provide verbal assent or if their parents or guardians did not consent to their participation.

### Sample size

The expected prevalence (p) was 0.0003 from the result of stool tests by JAPC in 21 prefectures in Japan in 2010 [45]. The desired level of absolute precision (d) was set at 0.0015. The necessary sample size without considering the design effect (DEEF) was calculated, as follows: N = 1.96^2^ × p (1 − p) × (DEFF)/d^2^ = 512. Sampling in five clusters (prefectures) results in multiplication of the number by DEEF: DEFF = 1 + (n − 1) × ICC, where *n* is the average cluster size (102), and ICC is the intra-cluster correlation. ICC was calculated as 0.00068 from the same data [45] using the formula described by Ridout et al. [46], and DEFF was estimated at 1.07. Therefore, the necessary sample size was determined as 547. With no positive sample, the estimated upper confidence limit of the prevalence was [1 − 0.05^(1/547)^] × DEFF = 0.0058 (0.58%) [47].

### Sampling process

Briefing sessions for recruitment were held at the selected elementary schools, kindergartens, and the nursery wherever permitted by school leadership. In cases where in-person briefings could not be performed, the classroom teachers provided written recruitment materials to the child caregivers. During both forms of recruitment, consent forms and STH educational leaflets were distributed to all potential participants. Consent forms and questionnaires were collected prior to or at the time of sample collection. Consented participants were provided screw-capped tubes with collection spoons to provide a 150- to 200-mg stool sample. Participants were instructed to collect the first morning stool of the day and bring the sample to the collection point on site the same day during two or three pre-specified dates for sample collection. During sample collection at each site, samples were stored in an icebox filled with dry ice. Incentives were prepared for some schools. For the three elementary schools in Ehime, we offered a Japanese workbook to the participants. In one kindergarten in Nagasaki, one in Tochigi, and the nursery in Ibaraki, participants received an STH coloring sheet and cartoon stool stickers.

### Questionnaire

A questionnaire was administered to all participants to characterize demographic profiles, including participant age and gender, and primary STH risk factors, including access to WASH resources. The questionnaire sought information on the main source of drinking water, sanitation (availability of a sewage system and toilet facility at home), and hand-washing habits. Additionally, it included questions regarding potential environmental risk factors such as consumption of vegetables grown in home gardens and travel history within two years to countries where STH is endemic. The questionnaire included multiple-choice questions with space for expanded description wherever required. Because the participants were young children, caregivers of participants completed the questionnaire instead.

### Sample shipment

Upon receipt at Nagasaki University, 1 ml of 90% ethanol was added to each sample, and samples were stored in a freezer until transport to Smith College (Northampton, MA, USA) for molecular analysis. A commercial cooling system was used to maintain temperature control during transit (Nano cool 48–92 h, from 2–8 °C). All samples were processed and analyzed at Smith College, except for a single sample that was inadvertently excluded from the shipment. The DNA from this sample was extracted at Nagasaki University (Nagasaki, Japan).

### DNA extraction

DNA was extracted from approximately 50 mg of each fecal sample using the MP FastDNA spin kit for soil and the FastPrep-24 5G bead-beater (MP Biomedicals, Santa Ana, CA, USA) according to previously described methods [35, 48]. An internal amplification control plasmid (FJ357008) [49] was added to the sample supernatant immediately after homogenization to ensure successful DNA extraction.

### Multi-parallel real-time PCR

Owing to its demonstrated ability to improve detection sensitivity and specificity, we used multi-parallel real-time PCR, targeting highly repetitive non-coding DNA sequences [35]. Multi-parallel real-time PCR (in individual 7-µl reaction volumes) was performed on all samples, as described previously [35], to detect DNA of *A. lumbricoides*, *N. americanus*, *A. duodenale* and *T. trichiura*. Testing was performed using the StepOne Plus instrument (Applied Biosystems, Foster City, CA, USA), and all samples were tested in duplicate. For positive-control purposes, for each assay, a combination of genomic DNA (gDNA) and plasmids containing a single-copy target was used. All gDNA control stocks were prepared according to the “SWDNA1” protocol available on the Filarial Research Reagent Resource Center website (http://www.filariasiscenter.org/parasite-resources/Protocols/materials-1/), with control reactions performed containing 2 ng, 200 pg and 20 pg of gDNA. Single-copy plasmids were generated using the Zero Blunt Topo PCR cloning kit (Thermo Fisher Scientific, Waltham, MA, USA), and Sanger sequencing was used to verify target sequences. Plasmid controls were tested at concentrations of 20 pg, 200 fg and 2 fg per reaction. Both experimental and control reactions were considered positive if they amplified with a maximum Cq value of < 40 in both replicates.

## Results

### Study sites and population

Samples were collected in the prefectures of Nagasaki, Ehime, Kochi, Tochigi, and Ibaraki (Additional file 2: Figure S1) with a total of 12 schools participating. Two elementary schools and two kindergartens were located in Nagasaki, three elementary schools in Ehime, three elementary schools in Kochi, one kindergarten in Tochigi, and one nursery in Ibaraki (Additional file 1: Table S3). Briefing sessions for recruitment were held at four elementary schools, three kindergartens and one nursery. For the other four sites, written recruitment materials were provided to caregivers *via* classroom teachers.

**Fig. 2:**
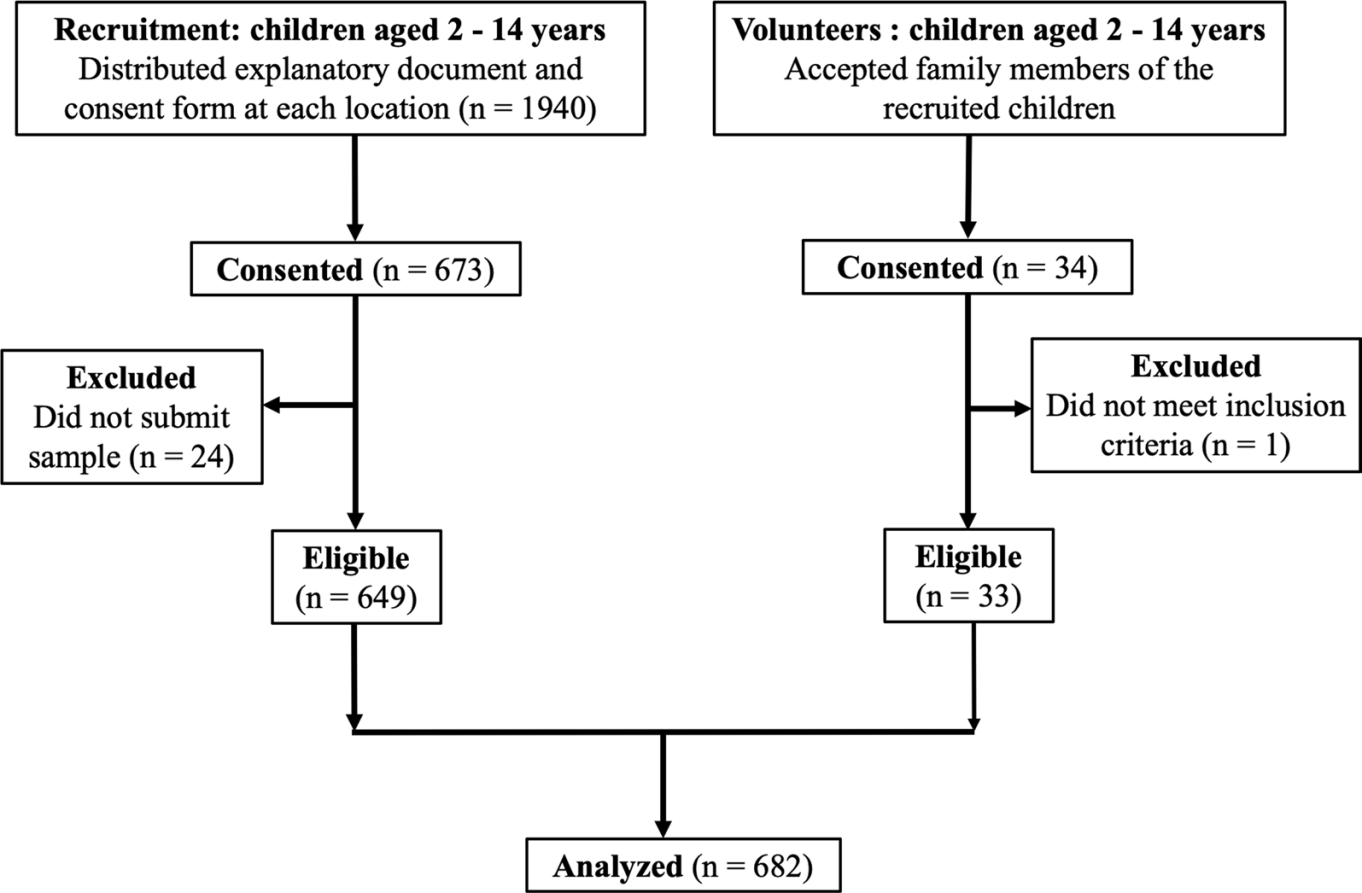
Sampling flowchart. Of the 1940 children recruited from elementary schools, kindergartens and a nursery, 649 submitted fecal samples. Additionally, family members meeting the inclusion criteria also voluntarily provided samples, of which 33 were eligible for analysis. In total, 682 samples were analyzed in this study

A total of 1940 children were initially recruited, of which 673 provided consent, and ultimately 649 stool samples were collected (Fig. 2). Additionally, some family members (aged 2–14 years) of the recruited children volunteered to participate in the study, resulting in collection of 34 samples. One sample from a preschool child was excluded due to age-related exclusion criteria. A single sample per participant was obtained [50–52] and, in total, 682 samples were analyzed in this study. This sample size provides the design effect of 1.09.

### Demographic profiles

Table 2 provides the demographic information of the participants. In total, 166 samples were obtained from preschool-aged children (24.3%) and 516 from school-aged children (75.7%). The gender ratio of the participants was 45.7% female and 54.3% male.

**Table 2:** Demographic information of participants from each location

Demographic information	Nagasaki (*N* = 236)	Ehime (*N* = 254)	Kochi (*N* = 81)	Tochigi (*N* = 96)	Ibaraki (*N* = 15)	Total (*N* = 682)
	*n* (%)	*n* (%)	*n* (%)	*n* (%)	*n* (%)	*n* (%)
Age group						
Preschool-aged children	67 (28.4)	0 (0.0)	2 (2.5)	86 (89.6)	11 (73.3)	166 (24.3)
School-aged children	169 (71.6)	254 (100.0)	79 (97.5)	10 (10.4)	4 (26.7)	516 (75.7)
Gender						
Female	98 (41.5)	122 (48.0)	40 (49.4)	44 (45.8)	8 (53.3)	312 (45.7)
Male	138 (58.5)	132 (52.0)	41 (50.6)	52 (54.2)	7 (46.7)	370 (54.3)

### Participation percentage

Survey participation at the elementary schools, kindergartens, and the nursery, ranged from 10.0% to 80.4% of the targeted populations (Additional file 1: Table S4). The overall participation percentage for fecal sample collection was 33.5% (Additional file 1: Table S4).

### Test results

Real-time PCR analysis of all fecal samples revealed no positive results for any species of STH (*A. lumbricoides*, *N. americanus*, *A. duodenale* or *T. trichiura*) among the 682 samples. The number of samples by prefecture is shown in Table 2. The internal amplification control was positive for all samples tested. Because no positive case was confirmed, the potential prevalence was calculated to be 0–0.48%.

### Questionnaire-based survey for investigating potential risk factors

The potential risk factors and behaviors obtained from 624 questionnaires are summarized in Table 3. One school in Kochi did not provide permission for the questionnaire to be administered; therefore, the 58 participants that provided stool samples were not surveyed using the questionnaire.

**Table 3:** Summary of potential risk factors and behaviors

Risk factor	Category	Total (*N *= 624)	
		*n*	(%)
Water			
Main source of drinking water	Piped into household or bottled water	605	(97.0)
	Well water	11	(1.8)
	No response	8	(1.3)
Sanitation			
Sewage system	Public sewage or septic tank	537	(86.1)
	Home treatment or other methods	16	(2.6)
	Unsure or no response	71	(11.4)
Toilet facility at home	Pit latrine	29	(4.6)
	Flush toilet or pour-flush toilet	577	(92.5)
	Unsure, no response, or multiple responses	18	(2.9)
Hygiene practice			
Hand-washing after defecation	Always or usually	538	(86.2)
	Sometimes or never	78	(12.5)
	No response	8	(1.3)
Hand-washing before eating	Always or usually	424	(67.9)
	Sometimes or never	194	(31.1)
	No response	6	(1.0)
Eating habits			
Consumption of homegrown vegetables	Yes	336	(53.8)
	No	261	(41.8)
	Unsure or no response	27	(4.3)
Travel history within 2 years			
To Asia, Africa and South America	Yes	19	(3.0)
	No	602	(96.5)
	No response	3	(0.5)

Regarding the main drinking water source, the vast majority (97.0%) of participants had a piped water supply or purchased bottled water. The percentage of participants drinking well water was low (1.8%). Most individuals reported access to either public sewage or a septic tank (86.1%), with only 2.6% treating sewage at home or *via* other methods. Additionally, most participants had flush or pour-flush toilets at home (92.5%). The percentage of pit latrine usage was 4.6%. Almost all respondents reported always or usually washing hands after defecation (86.2%), with most doing so before eating (67.9%).

Over half (53.8%) of the participants reported growing vegetables at home. Only 3.0% of the participants made overseas trips within the previous two years to countries in Asia, Africa, or South America, where STH might be prevalent. These results are presented by prefecture in Additional file 1: Table S5. The questionnaire was initially intended to identify correlates of STH infection; however, this was not possible, given that no positive cases were identified.

## Discussion

The failure to detect any STH infections in areas previously reporting high prevalence suggests that STH transmission has been interrupted in the sampled areas in Japan. Results were negative for all 682 fecal samples and for all species, indicating that none of the participants were infected by any of the four STH species. Prior STH-control programmes coupled with rapid economic development and improvements in infrastructure and policies are likely to have led to the elimination of STH transmission in Japan.

This is the first study to use multi-parallel real-time PCR, a highly sensitive and specific molecular-based diagnostic method [35, 53], for large-scale STH surveillance in Japan. Traditionally, the Kato’s cellophane thick smear technique has been used for large-scale helminth surveys in Japan [54]. Currently, the Kato-Katz method, an application of Kato’s cellophane thick smear technique, is recommended for STH surveys worldwide. Advantages of multi-parallel real-time PCR used in this study include the possibility of detection of positive samples without the mandatory presence of intact STH eggs in the stool sample. Even in the absence of intact eggs in the stool, 2 fg of helminth gDNA is sufficient for positive detection [35]. This method permits accurate determination of STH prevalence, even in areas with potentially very low transmission intensity.

There were no STH-infected individuals identified in the 12 facilities across the five prefectures where fecal testing was conducted in this study (Additional file 2: Figure S1). However, in these prefectures, isolated cases of STH continue to be documented (Additional file 1: Tables S1, S2). Prior to the 1994 abolition of the Parasitosis Prevention Law, several STH cases were traced to vegetables fertilized with night soil [55–58]. Although the use of night soil was suspected to be associated with STH infection, even after the abolition of this law, the Japan Agricultural Standards (JAS) for organic agricultural products were established in 2000 based on the Act on Japanese Agricultural Standards (JAS law). This law essentially banned the sale of organic agricultural products grown with human night soil. This possibly contributed to further declines in the prevalence of STHs [59].

Incident cases of STH infections in Japan are likely imported from STH-endemic settings *via* international travel or imported foods. The Tokyo Health Service Association also conducts screenings for parasitic infections of Japanese individuals who return to Japan after traveling abroad for > 6 months in accordance with the Industrial Safety and Health Act. These screenings detected STH eggs in 20 individuals between 2012 and 2016 [30]. Although 3.0% of the respondents to the questionnaire survey used for this study had travelled to Asia, South America, or Africa within the previous two years, there were no STH-positive individuals among them. Infection *via* imported vegetables [31] and processed food [32] is another possible route of infection; however, the Plant Protection Law bans import of plants with soil. Because STH and STH eggs should have been removed from produce prior to their being purchased by consumers, the risks associated with imported vegetables as sources of infection are extremely low. Furthermore, the Ministry of Health, Labour and Welfare issued notices to importers to reinforce guidance on prevention of STH infection *via* imported processed food products in 2005, when *Ascaris* eggs were detected in imported kimchi [60–62]. These notices were issued based on the Imported Foods Monitoring and Guidance Plan under the Food Sanitation Law.

The sustained elimination of STHs in Japan has been possible because of infrastructure, such as the water lines established during the postwar Japanese economic development, and comprehensive government policies. This study surveyed the socioeconomic and infrastructural status of the sampled population according to WASH standards. The WHO emphasizes the critical importance of activities that promote WASH as measures against neglected tropical diseases, including STHs [63]. The importance of WASH in controlling STHs was exemplified by a survey on low-income households in regions without proper wastewater treatment in the southern USA, which reported a 34.5% prevalence of hookworm infection in this specific population [34]. According to public sources, 90.9% of Japan is covered by wastewater treatment services including public sewage and septic tanks [64]. In the areas surveyed in the present study, wastewater was drained to the sewer or properly treated in a septic tank in 86.1% of homes. Furthermore, 92.5% of the population used flush toilets or simplified flush toilets. Additionally, access to tap water has been associated with reductions in the prevalence of *A. lumbricoides* and *T. trichiura* infection [65]. In Japan, 98% of the population has access to tap water [66] and 97.0% of the respondents in the present survey reporting drinking tap or bottled water.

STH disproportionately affects children of low socioeconomic status, immigrants, refugees, and adoptees from foreign countries [67]. By contrast, the proportion of immigrants among the total Japanese population is quite low (1.8%) [68], and Japan has very high standards of WASH. Therefore, regardless of whether the isolated cases are caused by persons who (re)enter Japan after being infected abroad [27–29] or through consumption of imported food [32], infections remain contained within the few infected individuals and are promptly resolved before spreading to surrounding communities or populations.

This study has several limitations. First, the prefecture-level estimates of prevalence from 1966 that were used to select participating schools might not be indicative of school-level prevalence. Additionally, the sample size of the included individuals was low (*n* = 682), and the 95% confidence interval included a fairly wide range of potential prevalence estimates (0–0.48%) [47]. This sample size allowed us to reasonably conclude that the prevalence was not above the elimination threshold at which transmission is likely to be sustained (1–2% prevalence by PCR) [69–71].

Another limitation is the highly variable and often low percentage of survey participation [72], which ranged between 10.0–80.4% (Additional file 1: Table S4). This may have led to considerable selection bias in those who participated, and the poor participation could have reduced the reliability of the survey results [72, 73].

Settings in which residents do not perceive themselves as at a risk of STH infection, such as in the localities surveyed in this study, could experience lower stool test participation during future STH surveillance activities. Additionally, negative perceptions surrounding the provision of stool tests, reluctance of handling stools, as well as embarrassment, all reportedly reduce stool-sampling participation [74]. Therefore, it is important to design the target sample size appropriately by estimating a non-response rate. In areas where verification of lymphatic filariasis (LF)-transmission interruption is ongoing, guidelines for the integrated transmission assessment of LF and STH among children indicate that refusals might be as high as 20% [75]. However, we found the non-response rate for STH surveillance in the Japanese post-elimination setting to be much higher. This may have important implications for the planning of future STH-surveillance activities in areas believed to have achieved transmission interruption.

## Conclusions

Among 682 individuals living in areas of Japan, previously endemic for STH infection, we did not detect any STH infection, suggesting that there is likely no STH recrudescence in the sampled areas. It appears that prior efforts to reduce STH transmission have likely resulted in sustained elimination of these infections. Although occasional STH infections continue to be introduced into Japan *via* individuals infected abroad or *via* imported processed food products, it is unlikely that these reach sufficient numbers to reestablish STH transmission within the population. This study suggests that STH elimination is possible and can be documented in areas where STH prevalence was previously reported as high. As numerous other countries move to consider a transition from STH control to elimination in focal geographic areas, documentation of what elimination actually can look like may be useful to policy makers seeking to implement changes in STH programmes. Furthermore, in addition to utility in settings with moderate to high prevalence [48, 76, 77], these data suggest that highly sensitive molecular techniques might also be useful for monitoring prevalence in areas where infection status is presumed to be quite low, as previously suggested [78], and can effectively rule out the presence of low-intensity infections that may pose a threat to sustained elimination. Continued economic development and application of governmental policies coupled with the expansion and improvement of MDA programmes for STH make the possibility of successfully moving from STH control to elimination an intriguing possibility.

## Supplementary information

**Additional file 1: Table S1.** Number of detected STH cases at each branch of BML, Inc. (courtesy of BML).** Table S2.** Number of detected STH cases at each branch of JAPC [37–45].** Table S3.** Study sites and their historic levels of STH.** Table S4.** Participation percentage of each school, kindergarten and a nursery.** Table S5.** Potential risk factors and behaviors per location.

**Additional file 2: Figure S1.** Map of Japan indicating the sample collection sites.

## Data Availability

Data supporting the conclusions of this article are included within the article and its additional files. The datasets analyzed in the present study are available from the first author upon reasonable request.

## Abbreviations

STH: soil-transmitted helminth
WASH: water, sanitation and hygiene
WHO: World Health Organization
MDA: mass drug administration
JAPC: Japan Association of Parasite Control
DEEF: design effect
ICC: intra-cluster correlation
gDNA: genomic DNA
LF: lymphatic filariasis
