# Supplementary material for: What does soil-transmitted helminth elimination look like? Results from a targeted molecular detection survey in Japan
Source: Parasit Vectors. 2020 Jan 8;13:6. doi: 10.1186/s13071-019-3875-z (PMC6950881; doi:10.1186/s13071-019-3875-z)
Supplement: Supplementary file 2 — Additional file 2: Figure S1. Map of Japan indicating the sample collection sites. [file 13071_2019_3875_MOESM2_ESM.pdf]

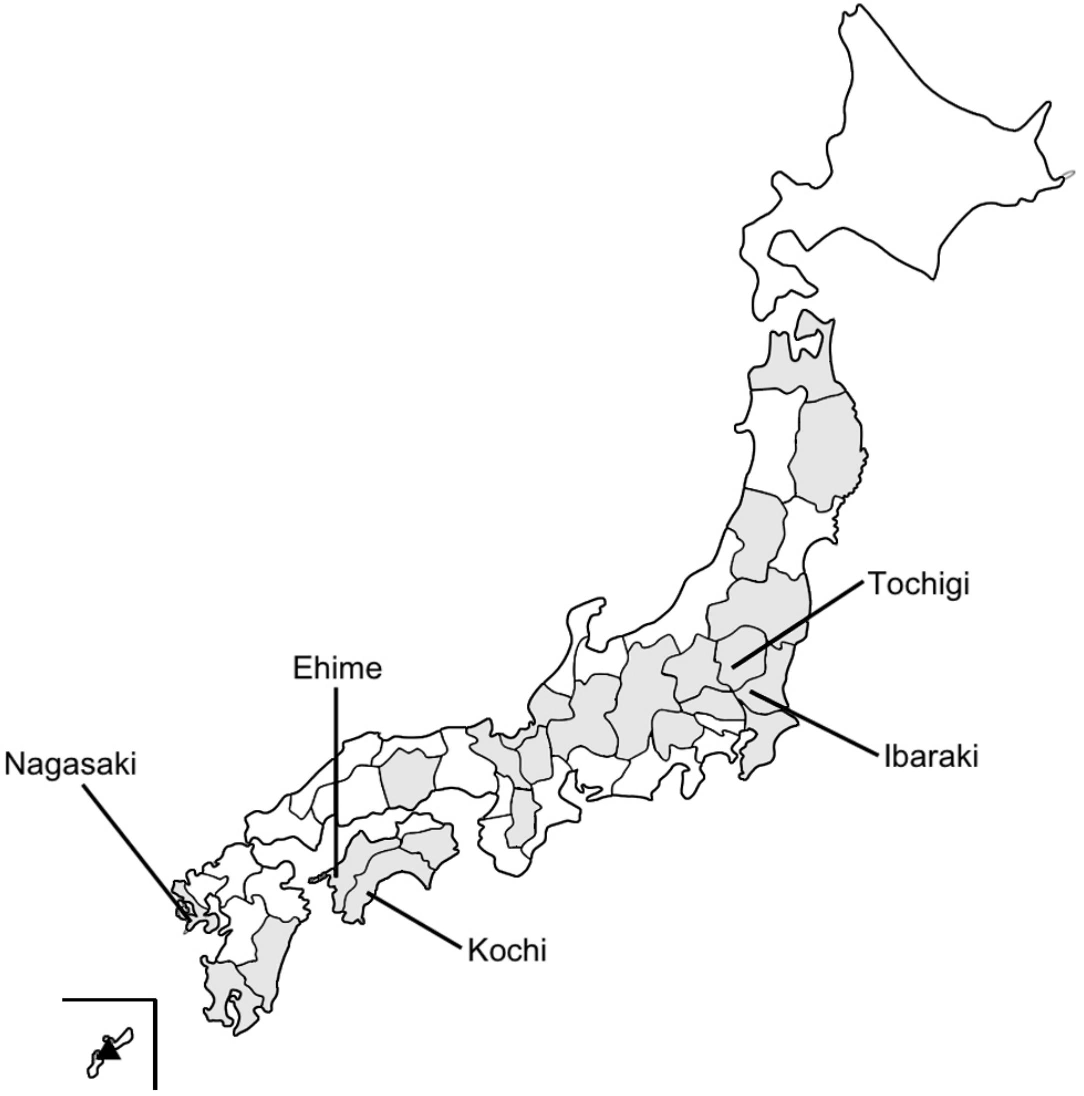

Additional file 2: Figure S1. Map of Japan indicating the sample collection sites.
Fecal samples were collected from six localities in five prefectures, namely Tochigi, Ibaraki, Ehime, and Kochi (one locality in each prefecture), as well as Nagasaki (two localities in this prefecture). These were included in the 23 prefectures that are colored grey, where the *Ascaris* egg-positive percentage was > 3.0% among elementary school children in 1966 with at least one confirmed STH case within a 15-year time-frame from 2002 to 2016. ▲: No data
